# Dislocation network with pair-coupling structure in {111} γ/γ′ interface of Ni-based single crystal superalloy

**DOI:** 10.1038/srep29941

**Published:** 2016-08-11

**Authors:** Yi Ru, Shusuo Li, Jian Zhou, Yanling Pei, Hui Wang, Shengkai Gong, Huibin Xu

**Affiliations:** 1 School of Materials Science and Engineering, Beihang University, No. 37 Xueyuan Road, Beijing, 100191, PR China

## Abstract

The γ/γ′ interface dislocation network is reported to improve the high temperature creep resistance of single crystal superalloys and is usually found to deposit in {001} interface. In this work, a new type of dislocation network was found in {111} γ/γ′ interface at a single crystal model superalloy crept at 1100 °C/100 MPa. The dislocations in the network are screw with Burgers vectors of 1/2 a<110> and most interestingly, they exhibit a pair-coupling structure. Further investigation indicates that the formation of {111} interface dislocation network occurs when the γ′ raft structure begins to degrade by the dislocations cutting into the rafted γ′ through the interface. In this condition, the pair-coupling structure is established by the dislocations gliding in a single {111} plane of γ′, in order to remove the anti-phase boundary in γ′; these dislocations also act as diffusion channels for dissolving of the γ′ particle that is unstable under the interfacial stress from lattice misfit, which leads to the formation of {111}-type zigzag interface. The formation of this network arises as a consequence of more negative misfit, low-alloying γ′ particle and proper test conditions of temperature and stress.

Ni-based single crystal superalloys have been widely used for turbine blades in most advanced aero-engines because of their excellent high temperature creep resistance^1^,^2^. This material is composed of a face-centered cubic Ni matrix (γ) and coherently precipitated L1_2_-structure Ni_3_Al cubes (γ′)^3^. The difference between the lattice parameters of γ and γ′ produces the lattice misfit and associated stress field in γ/γ′ interface^4^. To relax the misfit stress, a dislocation network in the interface has formed prevalently during high temperature creep^5^,^6^,^7^,^8^,^9^,^10^ and reasonably plays an important role in creep property^5^,^6^,^11^,^12^.

Many investigations have been carried out to interpret the formation of the network. When the lattice misfit is −0.3~0.3%, the dislocation network in (001) interface is composed of two kinds of edge dislocations which are (Burgers vector 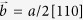, line vector 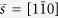) and (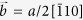, 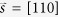)^5^,^7^,^8^,^10^. The misfit becomes lower than −0.6% in 4^th^ generation superalloys such as TMS-138^11^,^12^,^13^ which contains more Re and Ru. The configuration of the edge dislocations in (001) interface network significantly changes to (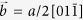, 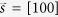) and (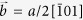, 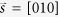) as a consequence of the more negative misfit.

The γ-γ′ interface of {001} type is prevalent in the γ′ raft structure which is completed during steady-state creep at high temperature. However, the {111}-type distorted interface has been found frequently when the γ′ raft structure continues to be broken by dislocations cutting via {111} plane of this interface; this phenomenon is prevalent in superalloys crept at high temperature/low stress^5^,^14^,^15^,^16^. In addition, as the misfit becomes more negative, the stress field in the interface increases^4^; this leads to the dissolution of the unstable γ′ phase near the interface^17^, which contributes to the formation of {111} interface. Based on the model in the previous works^5^,^18^,^19^,^20^, creep deformation of alloys can be ascribed to the dislocations gliding/climbing along the γ/γ′ interface which evolves into {111} type during degrading of the raft structure. Therefore, there is a critical issue concerning whether the dislocation network is able to establish in {111} interface. The dislocation network in {111} interface has been studied in only few works^4^,^21^,^22^. However, there is still debate in the type of dislocations in the network: being 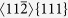 in simulation results^21^,^22^, while 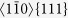 in CMSX-3 superalloy crept at 850 °C^4^, as the detailed investigation of {111} interface dislocation network has not been attempted.

Here we report the configuration of the {111} interface dislocation network in a single crystal model superalloy. After stress ruptured at 1100 °C/100 MPa, a new type of dislocation network in the {111}-type zigzag interface was found. The dislocations in the network are screw type and most interestingly, they exhibit the pair-coupling structure. Further investigation is carried out to reveal the formation mechanism of this network. Its formation is associated with degrading of the γ′ raft by dislocations cutting into γ′ phase through the interface. The factors influencing the formation are also discussed, such as the lattice misfit, the alloying design and the test conditions.

## Results

**Figure 1:**
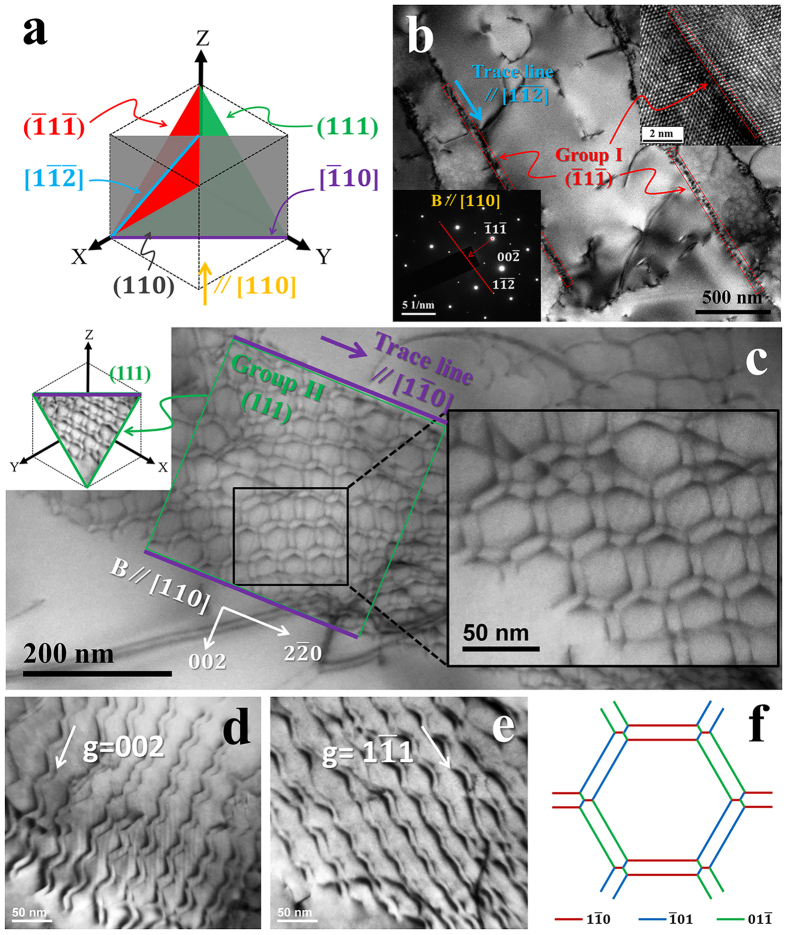
Observations of the {111} interfaces and associated dislocation network in the specimen ruptured at 1100 °C/100 MPa. (**a**) Observation of {111} interfaces with the electron beam incidence parallel to [110] (B//[110]); (**b**) TEM and High-Resolution TEM images of (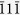) interfaces; (**c**) TEM image of the (111) interface dislocation network composed of coupled dislocations; the bright-field TEM images of the network (**d**) using **g** = 002 and (**e**) using **g** = 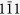; (**f**) Burgers vectors of the dislocations in the network, showing all the dislocations are screw.

Prior to observing the ruptured specimen, the configuration of {111} planes using TEM with the electron beam parallel to [110] direction (B//[110]) is illustrated in Fig. 1a. It is found that these {111} planes can be divided into two groups, namely Group I and II according to whether the plane is parallel to [110] electron beam incidence; in this condition the {111} plane in Group I or II is edge on or tilted respectively. The defined characterization of these planes is summarized in Table 1. For comparison the configuration of {001} planes in the same condition (B//[110]) is also listed in Table 1.

Both groups of the {111} interfaces in specimen ruptured at 1100 °C/100 MPa were experimentally observed and investigated in details. In Fig. 1b, a substantial amount of γ/γ′ interfaces were found to be edge on. The characteristics can be summarized as following: they were parallel to the electron beam incidence and perpendicular to 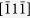 direction; their trace line was in 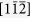 direction. As expected in Table 1, only the 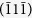 interface from Group I can exhibit such characteristics. This indicates that the formation of {111} interfaces occurs during 1100 °C stresses rupture test.

Observation of the tilted {111} interface is necessary to confirm whether dislocation network is able to establish in {111} interface. Figure 1c showed a tilted (111) interface with the trace line parallel to 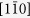 direction, as Group II in Table 1. It is interestingly found that a new kind of dislocation network has formed in this interface. This regular dislocation network was standard hexagonal and most significantly, each side was composed of a couple of dislocations. The technology of two beam diffraction and standard contrast extinction rules was used to investigate the dislocations’ Burgers vectors. For example, Fig. 1d,e show invisibilities of the parallel coupled dislocations using different g-vectors. It is determined that three kinds of coupled dislocations which formed the sides of hexagon were (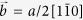, 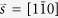), (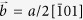, 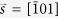) and (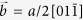, 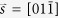), see the red, blue and green lines in Fig. 1f; all the dislocations were completely screw. These experimental results indicate that this network differs significantly from not only the general {001} interface networks with edge dislocations^11^,^12^,^13^ but also the reported {111} interface networks without pair-coupling structure^4^,^21^,^22^.

For a dislocation network in {001} interface, the dislocation spacing d varies inversely with the absolute value of misfit |δ| by an equation |δ| = 0.254/d^23^,^24^. However, this is obviously not suitable for the {111} interface dislocation network due to its varied spacing of dislocations with pair-coupling structure. Thus the density of dislocation in network, the average dislocation length per area of an interface, is used to quantify dislocation compactness.

**Figure 2:**
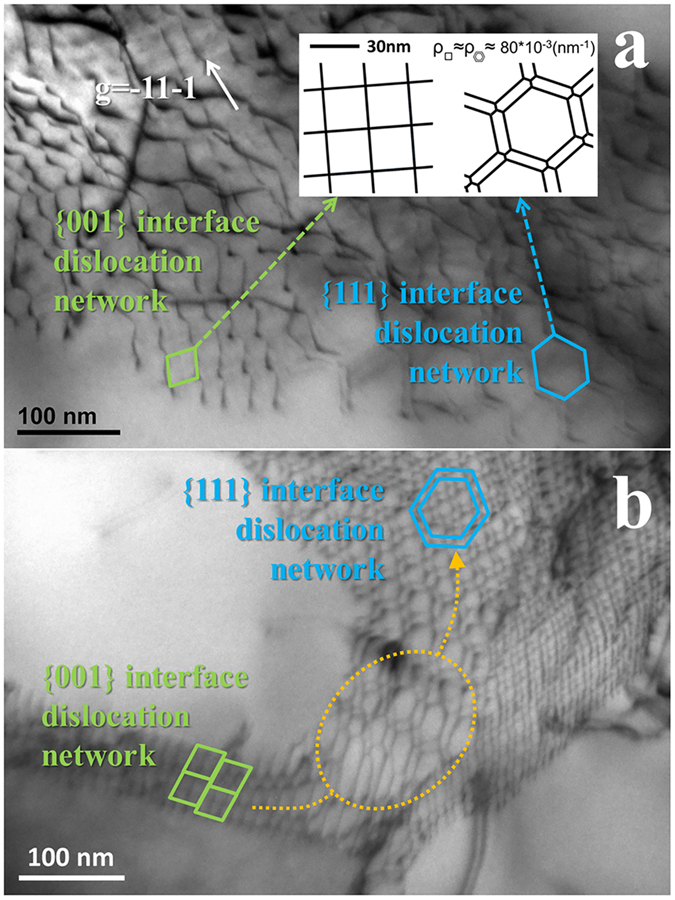
TEM images of {111} interface dislocation network that is accompanied with the squared network. (**a**) Dislocation density of both types of networks was similar in spite of different configuration; (**b**) the dislocations configuration transformation from {001} interface network to {111} ones.

From Fig. 2a, a {111} interface dislocation network (in the right of picture) was found to be accompanied with the {001} interface network which is square in shape (in the left); their dislocation densities were approximately (7.8 ± 0.5)*10^−2^ nm^−1^, almost the same to each other. The dislocation configuration transformation from {001} interface dislocation network to {111} one was also observed in Fig. 2b. It is found that the 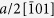 and 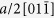 dislocations in the transformation region (the orange area) were intact and continuous; this is reasonable since the two kinds of dislocations with Burgers vector 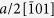 and 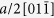 were found to establish both the {111} interface network (Fig. 1f) and the {001} one.

Although these dislocations have the same Burger vectors in {001} and {111} interface, the characters are quite different. From the observation of Fig. 1, the dislocations in {111} interface network is completely screw, which differs from the edge type of dislocations in {001} interface network. The stress from lattice misfit is not completely released by the {111} interface dislocation network due to its screw type. Link *et al*. have investigated the normalized energy of the network composed of edge/mixed dislocations with different Burgers vectors during creep at 1100 °C in second-generation SC superalloy CMSX-4; it is demonstrated that all the dislocations, whether they are 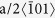, 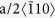 or 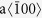, are prone to becoming edge type in order to completely release the misfit stress and thus reduce the total energy^25^. This is supported further by the simulation results that the dislocations in network exhibit edge type in the (100), (110) or (111) types of interface^21^,^22^. But the dislocation in {111} interface network is found to be completely screw as shown in Fig. 1f. The screw character of the dislocations in {111} interface indicates the stress field from lattice misfit cannot be completely released.

**Figure 3:**
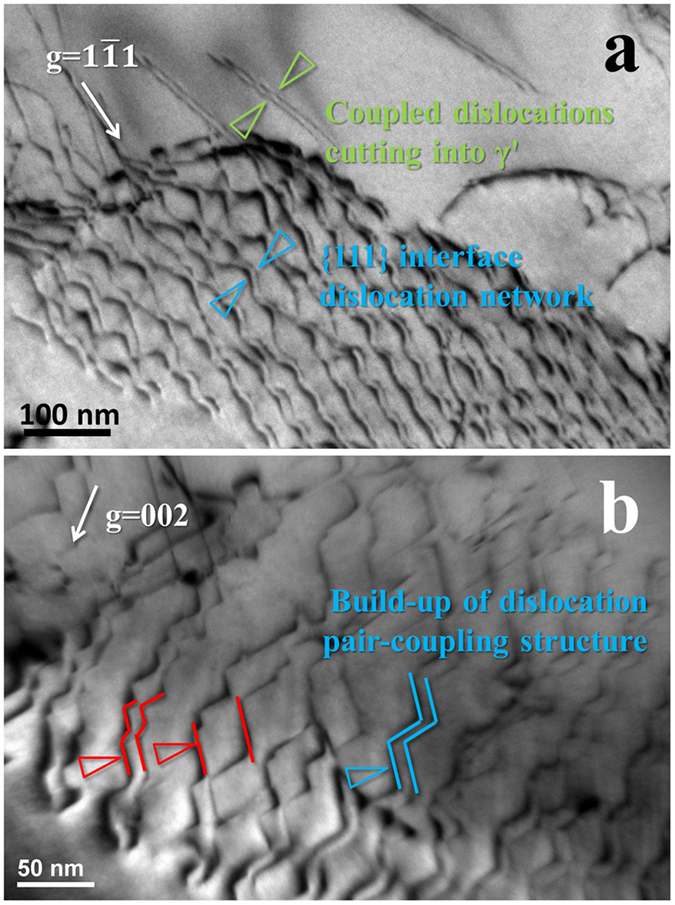
Observation of the pair-coupling structure and its forming process. (**a**) Coupled dislocations in {111} interface network and ones cutting into γ′, both with similar configuration; (**b**) forming process of the pair-coupling structure in the network.

Formation of the pair-coupling structure in {111} interface dislocation network was further studied. Figure 3a shows the comparison between the coupled dislocations in network and the ones cutting into γ′; it is found that they had comparable characteristics and a similar spacing in dislocation pair. Based on previous studies^26^,^27^,^28^,^29^, the dislocations gliding in a single {111} plane of γ′ phase are prone to couple with each other in order to remove the energy of anti-phase boundary (APB) in L1_2_-structure ordered Ni_3_Al. This formation mechanism of coupled dislocations might be extended to the pair-coupling structure in the network; the observation in Fig. 3b supports this suggestion. It is found that the neighboring dislocations exhibiting same line vectors and Burgers vectors glided closer from the red region to the blue one of Fig. 3b, with the pair-coupling structure eventually established; their line vectors and Burgers vectors does not change in this process. This process arises as the screw dislocation of 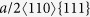 form is easy to glide in {111} planes under applied stress.

**Figure 4:**
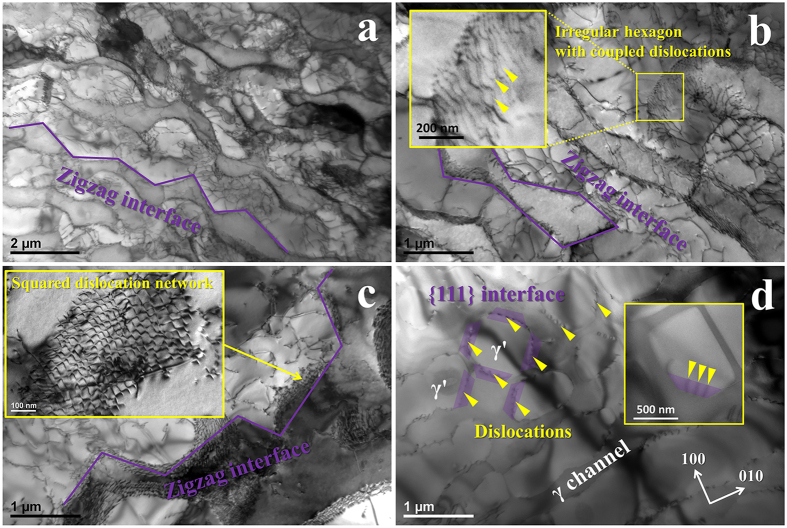
TEM observations of the γ/γ′ interfaces and dislocation configurations deposited in the interface in different testing conditions. Microstructure images of the specimens ruptured at (**a**) 1100 °C/100 MPa and (**b**) 1100 °C/130 MPa, showing the presence of {111} interface dislocation network and the pair-coupling dislocation structure; the network with coupled dislocations was not found in the specimen ruptured at (**c**) 980 °C/250 MPa; (**d**) after isothermally exposed of 1100 °C/100 h, the formation of {111} interface was associated with the dislocations moving in the interface.

The study about the formation of {111} interface dislocation network under conditions of temperatures and stresses has been conducted in Fig. 4. Figure 4a is the low magnification TEM image of the specimen ruptured at 1100 °C/100 MPa which has been carefully investigated above. The interface was found to become zigzag as a result of the dislocation cutting into γ′ through the interface during creep deformation; the {111} interface dislocation networks were formed in this regime. After 1100 °C/130 MPa crept, the zigzag interface was also found in Fig. 4b; more significantly, the dislocations deposited in interface exhibited irregular hexagonal shape with pair-coupling structure. Irregularity of this network presumably arises because of the short rupture life which is not sufficient to establish the complete network. Figure 4c shows the γ/γ′ microstructure after 980 °C/250 MPa stress rupture test. Although the interface was zigzag, there existed only square dislocation network, see the inset of Fig. 4c. A considerable amount of {111} interfaces formed in the specimen which was isothermally exposed at 1100 °C for 100 h, as shown in Fig. 4d. It is found that these interfaces were covered with dislocations; this indicates that the formation of {111} interface is associated with the dislocations moving in interface. However, the {111} interface dislocation network with pair-coupling structure was not observed. Therefore, it is confirmed that the sufficiently high creep temperature and applied stress are required for establishing the {111} interface dislocation networks.

## Discussions

The effect of the testing condition on the formation of {111} interface dislocation network is studied as summarized in Table 2; these experimental results indicate that the network formation arises as a consequence of both the applied stress and the creep temperature. Rafting of γ′ structure and its following degradation are prevalent for Ni based single-crystal superalloys during high temperature/ low stress creep^5^,^14^,^15^,^16^. Under the applied stress, the dislocations gliding from γ channel start to cut into the rafted γ′ during the degrading of rafting structure; at this point, they couple with each other due to the removal of APB in γ′. This is supported by the observation in Fig. 4d that the pair-coupling structure cannot form without the applied stress, as the dislocations move only in γ channel. The higher temperature accelerates the diffusion process that the γ′ phase near the distorted interface dissolves because of the misfit stress in interface; the dislocations gliding via {111} planes of the interface act as channels for diffusion of γ′ forming elements. Such that the zigzag {111} interfaces eventually form during this diffusion process.

**Figure 5:**
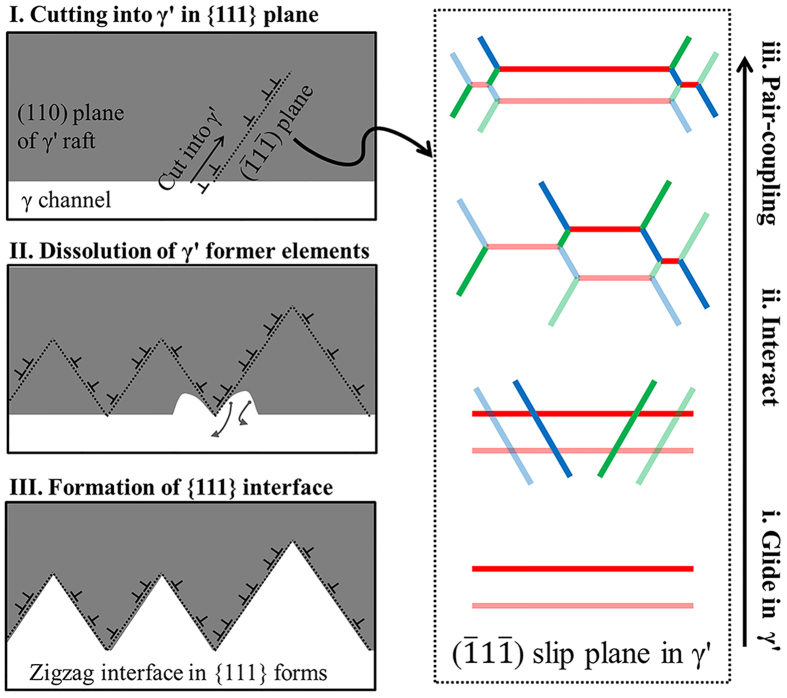
Model for the formation of the {111} interface dislocation network. Formation of {111} interface dislocation network occurs when the dislocations start to cut into the rafted γ′ through the {001} interface (see Stage I). The dislocations gliding in a {111} plane of γ′ phase become coupled to remove the APB (see Stage i→iii). The γ′ forming elements near to these dislocations continue to dissolve under the stress field induced by more negative misfit (see Stage II). Eventually in the Stage III, the network with coupled dislocations is completed in the zigzag {111} interface.

Figure 5 illustrates the formation mechanism of the {111} interface dislocation network. Rafting of γ′ structure is complete in the steady-state creep region. With creep deformation accumulating, the dislocations from γ channel cut into the rafted γ′ phase by gliding in {111} planes of γ/γ′ interface, as shown in the Stage I of Fig. 5. In this case, the configuration evolution of the dislocations gliding in a 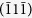 slip plane is shown in the magnified right part of Fig. 5. These screw dislocations with different Burgers vectors glide in this plane and interact with each other; the irregular hexagonal network is then established as shown in the Stage ii of Fig. 5, which is consistent with the red region of Fig. 3b. Thereafter, when it comes to the Stage iii, the pair-coupling dislocation structure with a critical spacing has formed in order to remove the APB energy; consequently, the hexagonal dislocation network with pair-coupling structure has established in the 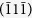 plane, the same to the configurations in Fig. 1. What occurs simultaneously with this network formation is the γ/γ′ interface evolution. In the Stage II of Fig. 5′ forming elements in the γ′ raft near to this network is diffusing to γ channel through numerous dislocations, since there exists the strong interface stress field induced by the negative misfit; this is supported by the experimental finding in Fig. 4d that the formation of {111} interfaces arises as a result of the same diffusion process. Eventually the {111} interfaces and associated dislocation networks have formed.

This dislocation network is quite different from the ones reported in previous works^4^,^21^,^22^. The molecular dynamics simulations^21^,^22^ found that the {111} interface dislocation network has been established by the edge dislocations with an index of 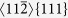, as these dislocations allow effectively relaxing of interfacial misfit stress; however, the dislocations in the network investigated in our work are of screw type with Burgers vectors of 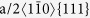. There are two reasons for the difference between experimental and simulated results. Firstly, the 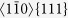 dislocation is more favored than 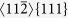 dislocations during 1100 °C creep deformation. Reed’s work^30^ confirmed that the macroscopic deformation of CMSX-4 varies with test condition of temperature and stress: being 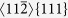 at 750 °C/750 MPa, while 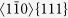 at 950 °C/185 MPa. At low temperature and high stress, the dislocations with Burgers vectors of 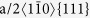 type react to form dislocations of 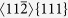 type accompanying with the superlattice intrinsic and extrinsic staking faults (SISF and SESF) as well as the anti-phase boundary (APB). Rae^31^ found that the 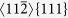 dislocations movement requires i) sufficient densities of 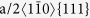 dislocations in γ and ii) sufficient resolved shear stress. On the contrary, in the conditions of high temperature and low stress the deformation is associated with the glide/climb of 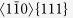
dislocation, i. e. octahedral slip, as supported further in the refs 4,32. Usually the deformation occurs in the form of a/2<110> dislocation pairs cutting into γ′ phase. Our experiment results indicate 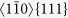 dislocations contribute to the deformation during 1100 °C/100 MPa creep test. Secondly, as shown in Fig. 4, the formation of {111} interface is caused by distorting of original {001} interface by the dislocations cutting into rafted γ′ via {111} planes; while these simulated networks were established in a given pure {111} interface that is free of the dislocations and their effect, which is not well consistent with the fact. Another experimental result about the {111} interface dislocation network has been studied in the work concerning the creep behavior of CMSX-3 at 850 °C^4^. These networks lie in {111} planes and were composed of three 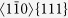 type dislocations, in agreement with our results; however the pair-coupling structure was not found. During the 850 °C creep, the γ′ particles remain as cubic except slightly dissolving in their corner; only a small amount of {111} interfaces forms in the corner. Under this condition, the formation of the interface dislocation network could be similar to the result of Fig. 4d; the pair-coupling structure is not able to establish without strongly shearing of the γ′ raft by dislocations.

Most significantly, the network formation is predominately ascribed to the alloying design that is characterized by increased addition of Mo and Al. Mo is an important γ-strengthening element due to its strong partition to γ, and its addition yields the lattice misfit of more negativity^11^,^33^,^34^,^35^. The spacing of dislocations in squared network of IC11B is determined as 29.4 + 2.7 nm, and hence the misfit in a {001} interface is calculated to be −0.86 ± 0.05%; this is significantly more negative than TMS-138^11^ and CMSX-3^4^. The more negative misfit induces the stronger stress field in the interface, in particular in the corner of γ′ cube^4^, and as a result, the corner dissolves to form {111} interfaces in Fig. 4d; this result can be extended to the γ′ raft degradation that the γ′ near to the distorted interface tends to dissolve under the stress field. Addition of extra Al and less Ta directly causes a considerable decrease in the Ta concentration in γ′ phase (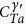). In addition to this, 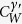 is equal to 0 since no W is applied into alloy. The composition of the rafted γ′ phase in IC11B is determined as (a.t. %) (13.46 ± 1.10)Al- (2.79 ± 0.76)Mo- (1.66 ± 0.55)Ta- (0.21 ± 0.08)Re- (2.49 ± 0.28)Cr- bal. Ni; there is a considerable decrease in 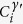 by comparison to the alloy ME-15^36^. Based on the work^4^, the threshold resolved shear stress 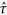 can be calculated by an equation of 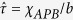, where 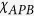 is the APB energy which is associated with 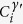; b, the magnitude of dislocation Burgers vector. IC11B with decreased 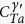 and 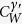 exhibits limited 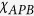, which means the resistance for the dislocations cutting into rafted γ′ is substantially reduced; this then results in a considerable amount of dislocations gliding in γ′ which is sufficient to establish the network.

## Conclusion

The γ/γ′ interface and associated dislocation network have been systematically investigated at a single crystal model superalloy in stress rupture test at 1100 °C/100 MPa. A new type of dislocation network in the {111} interface was found. The dislocations in network exhibit pair-coupling structure; their Burgers vectors are 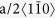, all of them being screw. The pair-coupling dislocation structure establishes in order to reduce the anti-phase boundary energy; the zigzag interface in {111} type forms as a consequence of the dissolution of γ′ phase through the dislocations which cut into the γ′ raft via {111} planes of interface. Further rupture tests indicate that sufficiently high creep temperature and applied stress are required for such network formation. The formation mechanism of the {111}-type zigzag interface and the dislocation network with pair-coupling structure is proposed; the alloying design for such network - in particular −0.86 ± 0.05% misfit and low-alloying γ′ particle - is also studied.

## Methods

### Alloying design for forming the {111} interface

In order to enable the formation of the {111} interface, the alloying composition of single crystal superalloy is carefully designed. Mo can confer more negative lattice misfit due to its strong partition to γ^11^,^33^,^34^,^35^; while W barely affects the misfit because of its partition to both γ and γ′^36^. Thus, substituting Mo for W is introduced at a model superalloy. Another alloying feature required for this work is the low alloying in γ′ which reduce the energy for shearing of rafted γ′ by the dislocations cutting. Both the Al increase and the Ta decrease can lead to the reduction in the Ta concentration of γ′, i.e. the lower solid solution strength of γ′. Consequently, the single crystal model superalloy IC11B is designed with a composition of (7.1~7.7)Al –7Mo –3Ta –1Re –2.2Cr –0.05Y –bal. Ni (w.t. %).

### Stress rupture test of the single crystal rods and observation using TEM

The single crystal rods were prepared by the as-cast process with screw selection crystal method, and then the standard heat treatment of 1320 °C/4 h –1340 °C/10 h –1120 °C/2 h –870 °C/24 h was carried out. After heat treatment, the segregation of elements between inter- and intra-dendrite was eliminated, and the γ′ particle was uniformly cubic with a size of 0.53 ± 0.06 μm. The orientations of all rods were determined by the indexing of back Laue patterns, and the rods with orientation of within 7° of the [001] crystallographic direction were chosen to be examined. The stress rupture tests were performed along [001] direction under different conditions. The stress rupture life of IC11B at 1100 °C/100 MPa is 108.07 h or 127.97 h; its (110)-foils were investigated in details using a transmission electron microscope (TEM) with the technology of two beam diffraction and standard contrast extinction rules. The foils of other specimens ruptured at 1100 °C/130 MPa and 980 °C/250 MPa were also observed. In addition, the microstructural evolution after isothermally exposed at 1100 °C/100 h and immediately water quenched was examined.

## Additional Information

**How to cite this article**: Ru, Y. *et al*. Dislocation network with pair-coupling structure in {111} γ/γ′ interface of Ni-based single crystal superalloy. *Sci. Rep*. **6**, 29941; doi: 10.1038/srep29941 (2016).

## Figures and Tables

**Table 1:** Characteristics of {111} and {001} interfaces observed by TEM using B//[110].

Interface	Group	Plane	Parallel to electron beam (B//[110])?	Edge on or not?	Trace lines in (110) plane
{111}	I	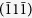 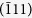	Yes	Edge on	// 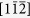 // 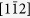
	II	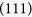 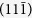	No	Tilted	// 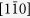
{001}	III	(001)	Yes	Edge on	// 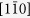
	IV	(100) (010)	No	Tilted	//[001]

**Table 2:** Formation of {111} interface and the associated dislocation network under conditions of stresses and temperatures.

Stress rupture test		Observation in TEM	
Temperature/Stress	Life (h)	{111} interface	Network with coupled dislocations
1100 °C/100 MPa	127.97	V(visible)	V
	108.07		
1100 °C/130 MPa	22.33	V	V
	23.97		
980 °C/250 MPa	93.07	V	I (invisible)
	81.07		
1100 °C/100 h (Exposure)		V	I
